# Nurse work environment: comparison between private and public hospitals

**DOI:** 10.31744/einstein_journal/2018AO4322

**Published:** 2018-10-30

**Authors:** Bárbara Sharon Maia Pires, Luana Zétula Fernandes de Oliveira, Cibele Leite Siqueira, Liliane Bauer Feldman, Ramon Antônio Oliveira, Renata Cristina Gasparino

**Affiliations:** 1 Departamento de Enfermagem, Pontifícia Universidade Católica de Minas Gerais, Poços de Caldas, MG, Brazil.; 2 Grupo de Trabalho de Segurança do Paciente, Conselho Regional de Enfermagem de São Paulo, São Paulo, SP, Brazil.; 3 Escola de Enfermagem, Universidade de São Paulo, São Paulo, SP, Brazil.; 4 Universidade Estadual de Campinas, Campinas, SP, Brazil.

**Keywords:** Health facility environment, Hospitals, private, Hospitals, public, Accreditation, Ambiente de instituições de saúde, Hospitais privados, Hospitais públicos, Acreditação

## Abstract

**Objective:**

To compare the characteristics of the work environment that enable the professional practice of nurses in private and public organizations.

**Methods:**

A quantitative, exploratory, cross-section study, carried out in four health organizations - one public and three private, with 188 registered nurses. Participants answered the Brazilian version of the Nursing Work Index − Revised, which aims to evaluate the presence of characteristics that favor the development of nursing activities through 15 items distributed into three subscales: autonomy, control over the practice setting and relationships with physicians. The measurement scale used is Likert, and lower scores represent better evaluation of the environment, *i.e.* , more favorable characteristics are present to assist the development of nursing activities.

**Results:**

The means of the responses of participants of private hospitals were smaller in all subscales of the instrument, as compared to those from public hospitals.

**Conclusion:**

The environment of private hospitals showed more favorable characteristics to the professional practice of registered nurses than the public hospital environment.

## RESUMO

**Objetivo:**

Comparar as características do ambiente de trabalho que favorecem a prática profissional do enfermeiro em instituições privadas e pública.

**Métodos:**

Estudo quantitativo, exploratório, transversal, realizado em quatro instituições de saúde, sendo uma pública e três privadas, com 188 enfermeiros. Os participantes responderam a versão brasileira do *Nursing Work Index* − *Revised* , que tem por objetivo avaliar a presença de características que favorecem o desenvolvimento das atividades do enfermeiro por meio de 15 itens distribuídos em três subescalas: autonomia, controle sobre o ambiente de trabalho e relações entre médicos. A escala de medida utilizada é do tipo Likert e menores pontuações representam melhor avaliação do ambiente, ou seja, mais características favoráveis estão presentes para auxiliar o desenvolvimento das atividades da enfermagem.

**Resultados:**

As médias das respostas dos participantes dos hospitais privados foram menores em todas as subescalas do instrumento, quando comparadas às do hospital público.

**Conclusão:**

Os ambientes dos hospitais privados demonstraram possuir mais características favoráveis à prática profissional do enfermeiro do que o ambiente do hospital público.

## INTRODUCTION

During the 1980s, in the United States, there was great concern with the lack of professional nurses at health organizations. This was evident by the presence of almost 100 thousand vacancies and more than 80% of hospitals with inadequate sizing of the nursing team, due to the incapacity of the organizations to attract and retain qualified professionals.^(^ ^1^ ^)^

The reasons for this were studied, and the likely causes of this dissatisfaction were listed. The incorporation of new technologies, the increased complexity, the fragmentation of care and the inconstant presence of the medical team created a situation in which the nurse assumed new responsibilities without, however, reaching due recognition of their authority in this process.^(^ ^1^ ^)^

Considering that the nursing team is fundamental for the care of patients and family members, the American Academy of Nursing, initiated a force task to examine the characteristics that precluded or facilitated nurses in developing their skills. From then on, “magnetic hospitals” have appeared, providing environments that favor the development of nursing activities. Consequently, they reach better results^(^ ^2^ ^)^ for patients (lower mortality rate, lower incidence of pressure lesions, greater satisfaction with the care received, and the presence of a more sound culture of safety),^(^ ^3^ ^-^ ^6^ ^)^ professionals (higher level of professional satisfaction and lower level of burnout),^(^ ^6^ ^,^ ^7^ ^)^ and organizations (decreased intention of leaving the job).^(^ ^6^ ^-^ ^7^ ^)^

In healthcare organizations, the nursing team is responsible for 95% of care that patients receive during their hospitalization.^(^ ^2^ ^)^ Thus, knowledge of the characteristics of the environment, such as the quality of the relations with the medical team, and the autonomy and control that nurses have in the resolution of problems that affect patient care, should be a priority of the managers who are concerned about the excellence of the institutional results.

Assessment of the environmental quality is necessary to base the managerial practice of nurses. The managers and the nurses themselves, as team leaders, should be able to identify the presence or absence of characteristics that favor the nursing professional practice, so that they might implement actions that contribute towards improving this environment, and consequently, the results.^(^ ^8^ ^)^

Threfore, the issues that guided this study were - are there differences between the environments in private and public hospitals? Is the environment of private hospitals better evaluated by nurses than that of public hospitals, since they face growing competitively of the market?

## OBJECTIVE

To compare the characteristics of the work environment that favors the nursing professional practice among private and public institutions.

## METHODS

This is a quantitative, cross-sectional, exploratory study carried out at four healthcare organizations in the interior of the state of Estado de Minas Gerais, chosen by convenience. Hospital 1 (H1) is public, with 154 beds and 52 nurses. Hospital 2 (H2) is private, accredited by the National Accreditation Organization (ONA) [ *Organização Nacional de Acreditação* ], with 86 beds and 34 nurses. Hospital 3 (H3), private, also accredited by ONA, with 123 beds and 103 nurses, and hospital 4 (H4), private, had 88 beds and 25 nurses.

Considering that the population was composed of merely 214 professionals, it was decided that no sampling procedure would be done and that all those who satisfied the following inclusion criteria would be included: be a registered nurses, not be on vacation or leave of absence, have at least 3 months of professional experience at the organization, and agree to participate in the study by signing the Informed Consent Form.

In this way, the final sample was composed of 188 registered nurses (87.8% of the population), since 15 (7.0%) were on vacation or leave of absence; 7 (3.3%) had worked for less than 3 months at the organizations, and 4 (1.9%) refused to participate.

For data collection, we used a chart for characterization of the sample and the Brazilian version of the Nursing Work Index − Revised (NWI-R).^(^ ^9^ ^,^ ^10^ ^)^ The chart for personal and professional characterization of the participants covered the following variables: sex, age, marital status, characteristics of the organization, time of experience in the profession, professional training, setting of work, time of work at the organization, and weekly work load.

To evaluate the characteristics of the environment, the Brazilian version of NWI-R was used, which contains 15 items distributed into three subscales: autonomy, control over the work environment, and relationships with physicians.^(^ ^10^ ^)^

The measurement scale used is Likert-type, which varies between 1 and 4 points, and the smaller the score the greater the presence of characteristics that favor the development of the nurse’s professional practice as a nurse. The scores for the subscales were obtained by the mean score of the individual’s responses, which can vary between 1 and 4 points.^(^ ^10^ ^)^

After authorization by the organizations, the data were collected from April to June 2016. A date was scheduled with the nurses so that the investigators could explain the study objectives and give the envelopes containing the instruments to be completed. The sealed envelopes were returned to the investigators soon after the meeting or at a previously agreed upon date.

The database was created using Microsoft Excel for Windows version 2010^®^ (Microsoft Corporation, Redmond, WA, USA) by two independent researchers, and verified by a third researcher.

For data analysis, tables of absolute and relative frequencies of the categorical variables were prepared, and the measurements of location and dispersion (mean, standard deviation and median) of the continuous variables were calculated. The comparison between hospitals and instrument subscales was made by means of the Kruskal-Wallis test, followed by Dunn’s post-test when significant differences were found.

The research was approved by the Research Ethics Committee of the university under protocol CAAE: 48615515.1.0000.5137. All ethical precepts that regulate research involving humans were respected.

## RESULTS

A total of 188 nurses participated in the study. Table 1 shows the variables of personal and professional characterization of the participants.

**Table 1:** Description of variables of personal and professional characterization of registered nurses

Variables	n (%)
Sex	
Female	166 (88.3)
Male	22 (11.7)
Age group, years	
21-24	24 (12.8)
25-35	125 (66.5)
36-44	28 (14.9)
45-60	11 (5.9)
Marital status	
Single	92 (48.9)
Married	89 (47.3)
Others	7 (3.7)
Characteristic of the institution	
Private	143 (76.1)
Public	45 (23.9)
Experience in the profession	
3 months-1 year	40 (21.3)
1-5 year	88 (46.8)
6 or more	60 (31.9)
Professional background	
Graduated	56 (29.8)
Specialization	125 (66.5)
Others	7 (3.7)
Setting	
Inpatients ward	31 (16.5)
Intensive care units	80 (42.6)
Urgency and emergency settings	21 (11.2)
Others	56 (29.8)
Time working for the organization, years	
1-5	136 (72.3)
6 or more	52 (27.7)
Weekly work load, hours	
40-44	71 (37.8)
45-55	93 (49.5)
56 or more	24 (12.8)

The comparisons of nurses’ perceptions from the different institutions as to the characteristics of the environment where they practiced can be seen on table 2 .

**Table 2:** Perception of nurses from the different institutions regarding characteristics of the environment that favor their professional practice

Subscales	n	Mean	SD	Median	p value
Autonomy					
H1	45	2.1	0.6	2.0	<0.0001
H2	30	1.5	0.3	1.5	
H3	92	2.1	0.6	2.0	
H4	21	1.9	0.5	2.0	
Control over the environment					
H1	45	2.4	0.6	2.4	0.0003
H2	30	1.8	0.4	1.9	
H3	92	2.2	0.7	2.3	
H4	21	2.0	0.5	2.0	
Relationships with physicians					
H1	45	2.4	0.6	2.3	0.0003
H2	30	1.8	0.4	1.7	
H3	92	2.1	0.6	2.2	
H4	21	2.1	0.6	2.0	

* p value: obtained by the Kruskal-Wallis test. SD: standard deviation; H: hospital.

Since there were significant differences among the organizations in all subscales, Dunn’s post-test was used to detect such differences. Hospital 2 presented with more favorable conditions for work than H1 and H3 ( Table 3 ), in all subscales of the instrument.

**Table 3:** Differences when comparing perception of professionals from different institutions about the practice environment

Subscales	Result*
Autonomy	
H1 *versus.* H2	Significant
H1 *versus* H3	Non-significant
H1 *versus* H4	Non-significant
H2 *versus* H3	Significant
H2 *versus* H4	Non-significant
H3 *versus* H4	Non-significant
Control over the environment	
H1 *versus* H2	Significant
H1 *versus* H3	Non-significant
H1 *versus* H4	Non-significant
H2 *versus* H3	Significant
H2 *versus* H4	Non-significant
H3 *versus* H4	Non-significant
Relationships with physicians	
H1 *versus* H2	Significant
H1 *versus* H3	Non-significant
H1 *versus* H4	Non-significant
H2 *versus* H3	Significant
H2 *versus* H4	Non-significant
H3 *versus* H4	Non-significant

* Dunn´s post-test.

In the individual analysis of the items, on table 4 , ten situations that favor the professional practice of nursing were highlighted, and showed the highest percentage of disagreement among the participants; that is, positive situations, but that were not present in the work environment, according to the perception of the nurses in this study.

**Table 4:** Items favorable to the nursing professional practice that are not present in the work environment, as per the perception of the nurses

Items	n (%)
The nurses working regularly and permanently together never have to cover another unit	117 (62.2)
Opportunity for the nurses to participate in administrative decisions	110 (58.5)
A satisfactory salary	107 (56.9)
The nursing team participates in the choice of new equipment	101 (53.7)
The nursing team receives support to advance in the professional career	92 (48.9)
An administration that listens and responds to the concerns of workers	92 (48.9)
Sufficient time and opportunity to discuss with other nurses the problems related to patient care	87 (46.3)
Sufficient team to perform the work	82 (43.7)
Each nursing unit determines its own rules and procedures	82 (43.6)
Acknowledgment and praise for a work well done	79 (42.0)

## DISCUSSION

Private hospitals demonstrated better performance when compared to the public hospital, in the subscales of control over the environment, and relations among physicians and nurses. In the subscale autonomy, despite private hospitals 2 and 4 also having demonstrated a better performance, H3 reached the same score as the public hospital. Hospital 2 had a better performance on all subscales when compared to H1 and H3.

In general, the best performance demonstrated by the majority of private hospitals was expected by the researchers due to the competitiveness of the market, which requires the constant review of the processes, so that the organizations are kept sustainable, that is, that they are able to provide excellence care for the lowest financial cost possible.^(^ ^11^ ^,^ ^12^ ^)^

The hospital accreditation present in H2 and in H3, or the search for it (a fact observed in H4) drives important changes for the organizations and keep themselves competitive on the market.^(^ ^13^ ^)^ These changes go beyond the operation and management of quality, and reach the top management. Perhaps this is one of the reasons for better performance of private hospitals, which already have a strategic view towards the reorganization of the work environment.^(^ ^11^ ^,^ ^12^ ^)^

Although certified is a driving force for improvement in a broad sense,^(^ ^14^ ^)^ it was noted that among accredited private hospitals and those applying for certification, there were no significant differences, demonstrating that the models of quality and processes should be continuously monitored, discussed, and implemented again.^(^ ^12^ ^)^

When analyzing the characteristics reported by nurses as not being present in their work environment, it was noted that three of them were related to shortage of staff (“The nurses working regularly and permanently together never have to cover another unit”, “Sufficient time and opportunity to discuss with other nurses the problems related to patient care”, and “Sufficient to perform the work”).

Work overload can be identified by extensive weekly work load completed by the study participants, and other inadequate conditions, favor physical and emotional exhaustion of the professionals, leading to increased absenteeism and work accidents.^(^ ^15^ ^)^ The *Conselho Nacional de Enfermagem* [Federal Nursing Council] establishes the minimal parameters to determine the number of professionals carrying out nursing activitie.^(^ ^16^ ^)^ However, one of the major challenges of administrative nurses is to comply with this legislations,^(^ ^17^ ^)^ since it directly affects care delivery.^(^ ^18^ ^)^

Still regarding the difficulties pointed out by nurses, it was possible to note that a considerable part of the situations is related to the empowerment of the category, and to restructuring of work processes (“Opportunity for the nurses to participate in administrative decisions,” “The nursing team participates in the choice of new equipment,” “Acknowledgment and praise for a work well done” and “An administration that listens and responds to the concerns of workers”). Thus, such situations can be strategically worked on, to guarantee a better working environment for nurses and consequently, better results,^(^ ^19^ ^)^ with no need for big financial investments on the part of the organization.

All items of NWI-R represent characteristics favorable to the development of nursing activities, including the item “Each nursing setting determines its own rules and procedures.” Perhaps, when this situation was mapped for the first time, in 1989, it represented the autonomy the settings had to determine their own processes. Nevertheless, currently, since the Standard Operating Procedure is acknowledged as a tool to guarantee quality and safety of procedures, the some NWI-R items can no longer be considered a positive aspect for the professional practice of nursing and should be interpreted with caution.

This investigation is important for demonstrating that, even in case of financial difficulties faced by the healthcare organizations, much can be done by the managers to assure a human capital more satisfied and committed to the quality of care delivered and safety of patients. The “magnetic hospitals”, which invest in the environment in which nurses carry out their activities, have guaranteed better quality in care delivery.^(^ ^19^ ^)^

As a limitation of this study, we point out the fact of its having assessed only one public institution and in one single city in inland state of Minas Gerais, which compromises the generalization of the results for the entire national reality. Future research, involving a larger number of public and private institutions, accredited and non-accredited, and in different states of Brazil, should be conducted, so that the differences might be better understood and serve as a base for changes, aiming to improve results of all healthcare organizations.

## CONCLUSION

The environment of private hospitals demonstrated more characteristics favorable to the nurses’ professional practice than that of public hospitals. It was also possible to detect some situations that can help managers implementing strategies, with the objective of improving characteristics of the environment in which nurses carry out their activities.
